# Extracellular Vesicle Analysis by Paper Spray Ionization Mass Spectrometry

**DOI:** 10.3390/metabo11050308

**Published:** 2021-05-11

**Authors:** Casey A. Chamberlain, Marguerite Hatch, Timothy J. Garrett

**Affiliations:** Department of Pathology, Immunology and Laboratory Medicine, University of Florida, Gainesville, FL 32610, USA; chamberlain.c@wustl.edu (C.A.C.); hatchma@pathology.ufl.edu (M.H.)

**Keywords:** paper spray ionization, metabolomics, mass spectrometry, extracellular vesicles, *Oxalobacter formigenes*

## Abstract

Paper spray ionization mass spectrometry (PSI-MS) is a direct MS analysis technique with several reported bacterial metabolomics applications. As with most MS-based bacterial studies, all currently reported PSI-MS bacterial analyses have focused on the chemical signatures of the cellular unit. One dimension of the bacterial metabolome that is often lost in such analyses is the exometabolome (extracellular metabolome), including secreted metabolites, lipids, and peptides. A key component of the bacterial exometabolome that is gaining increased attention in the microbiology and biomedical communities is extracellular vesicles (EVs). These excreted structures, produced by cells in all domains of life, contain a variety of biomolecules responsible for a wide array of cellular functions, thus representing a core component of the bacterial secreted metabolome. Although previously examined using other MS approaches, no reports currently exist for a PSI-MS analysis of bacterial EVs, nor EVs from any other organism (exosomes, ectosomes, etc.). PSI-MS holds unique analytical strengths over other commonly used MS platforms and could thus provide an advantageous approach to EV metabolomics. To address this, we report a novel application representing, to our knowledge, the first PSI-MS analysis of EVs from any organism (using the human gut resident *Oxalobacter formigenes* as the experimental model, a bacterium whose EVs were never previously investigated). In this report, we show how we isolated and purified EVs from bacterial culture supernatant by EV-specific affinity chromatography, confirmed and characterized these vesicles by nanoparticle tracking analysis, analyzed the EV isolate by PSI-MS, and identified a panel of EV-derived metabolites, lipids, and peptides. This work serves as a pioneering study in the field of MS-based EV analysis and provides a new, rapid, sensitive, and economical approach to EV metabolomics.

## 1. Introduction

### 1.1. Paper Spray Ionization and Bacterial Metabolomics

Paper spray ionization mass spectrometry (PSI-MS) is an ambient MS approach that typically involves the direct analysis of a relatively small volume of unextracted biological sample deposited onto paper [1]. PSI-MS offers several advantages over conventional liquid chromatography (LC)-MS approaches, including minimal sample volume, reduced or eliminated dependence on extraction or other sample preparation, and shortened analysis time [2]. Due to these analytical advantages, PSI-MS has been applied as a novel tool in a variety of fields of research, including medicine [3], homeland security [4,5], microbiology [6], environmental management [7], quality control [8], toxicology [9], and many more. One PSI-MS application of increasing interest is the analysis of bacteria [6,10,11]. Over the years, a wide variety of MS technologies have been used for bacterial analysis [12,13,14,15,16,17,18], with PSI-MS emerging with an initial demonstration of genus and species-level analytical differentiation [6], and more recently, strain-level differentiation [10]. In most microbiology-focused metabolomics experiments, bacterial cells are often separated from their conditioned medium matrix and washed prior to MS analysis [19,20]. Such experiments provide a chemical characterization of the cellular unit, but information regarding the secreted metabolome, including all extracellular bacterium-derived metabolites, lipids, and peptides, is lost. This dimension of the molecular profile, often referred to as the metabolic footprint [21], is valuable to a bacterial metabolomics experiment as many microbes produce compounds that are predominantly secreted that may not be detected and identified in a strictly cellular analysis [22]. Secreted metabolites, together with free metabolites in the extracellular environment acted upon by bacteria, are cumulatively referred to as the exometabolome [23]. In vivo, the bacterial exometabolome is a major factor of the microbiome-derived exposome [24], making its characterization imperative to understand both the bacterium itself and its host-microbe biochemical relationship.

### 1.2. Bacterial Extracellular Vesicles

A primary component of the bacterial exometabolome is extracellular vesicles (EVs), membranous structures produced and secreted by cells in all three domains of life (Prokarya, Eukarya, and Archaea) [25]. EVs are believed to be ubiquitously produced among bacteria and have been characterized in many different species, including both Gram-negatives and Gram-positives [26], as well as in certain “atypical” bacteria not described by the widely used Gram’s method classification [27]. For the purpose of this discussion, we focus on bacteria that fit the conventional categorization as “Gram-positive” and “Gram-negative” by the presence of one or two lipid bilayer membranes in the cell envelope, respectively. Both classes possess a plasma membrane enclosing the cytoplasm, but Gram-negatives have an additional lipid membrane, known as the outer membrane, which lies beyond the peptidoglycan layer and encloses the periplasm [28]. Bacterial EVs are produced by budding from these lipid membranes and are described by the specific membranes from which they form. Gram-negatives produce two distinct types of EVs: outer membrane vesicles (OMVs) and outer-inner membrane vesicles (O-IMVs) [29]. OMVs consist of periplasmic contents coated in an external lipid membrane layer resembling the outer bacterial membrane from which these vesicles bud from the cell [30]. O-IMVs have two membranes, first originating from the cytoplasm with a plasma membrane-like coat, and then gathering a second layer from the periplasm and outer lipid membrane [29]. It was originally believed that only Gram-negatives produced EVs, but three decades later, it was discovered that Gram-positives also produce secreted vesicles, simply termed EVs; however, the mechanism by which they pass through the relatively thick peptidoglycan layer outside the plasma membrane to be secreted from the cell remains poorly understood [26,31,32]. EVs are rich in metabolites, lipids, and proteins and serve as a core mechanism of bacterial extracellular biochemical transport, communication, defense, and survival [33]. There is significant biomedical interest in the analysis of EVs as it has recently been shown that bacteria use them, among many other functions, to transfer genetic material and enzymes providing antibiotic resistance to other microorganisms [34,35]. Hence, these vesicles represent an important and medically relevant dimension of the exometabolome.

### 1.3. PSI-MS: A New Platform for Extracellular Vesicle Analysis

Despite strong literature representation of MS studies focused on eukaryotic EVs, particularly exosomes [36,37,38,39,40,41], MS applications to bacterial EVs are relatively limited. Among the reported MS bacterial EV analyses, nearly all are LC-MS-based proteomics studies [42,43]. Consequently, the field of bacterial EV metabolomics, as well as the application of direct MS analysis to bacterial EVs, is scarcely investigated. Among the few studies that have used direct MS analysis to study bacterial EVs, most have used matrix-assisted laser desorption ionization (MALDI) [42,44,45]. As described earlier, PSI-MS offers key analytical advantages over other analysis platforms. In comparison to LC-MS, PSI-MS offers reduced analysis time, lack of sample preparation, and minimal solvent consumption [1,2]. Although MALDI shares these qualities of expedited analysis and solvent conservation over LC-MS, reproducibility in its performance is dependent on proper pre-analytical application of a matrix to the sample [46], an additional step in sample handling and potential source of technical error that is not required in PSI-MS. Despite its analytical advantages over other commonly used approaches, the use of PSI-MS to analyze bacterial EVs, or EVs from any organism for that matter, has never been reported. A PSI-MS-based methodology for the analysis of bacterial EVs could provide a new, rapid, sensitive, and economical approach to analyzing the bacterial exometabolome. Hence, in this report, we describe the first PSI-MS application to bacterial exometabolomics by demonstrating the generation, isolation, confirmation, and PSI-MS analysis of bacterial EVs. The experimental model for our study was *Oxalobacter formigenes* (*O. formigenes*), a commensal, Gram-negative resident of the human intestinal microbiome with significant interest in the impact of its secreted metabolome on human health [47,48,49,50,51]. *O. formigenes* has been suggested to produce and expel a secretagogue compound that potentially curtails the risk of calcium oxalate kidney stone disease by stimulating a net intestinal secretion of oxalate, a precursor and risk factor for stone formation, which theoretically reduces its concentration in circulation and renal excretion [47,48,52,53,54]. A secreted bioactive compound of this nature could be expected to be contained in and expelled via EVs, so investigating vesicles produced by *O. formigenes* is of potentially notable importance. Until now, vesicles derived from *O. formigenes* had never been previously confirmed nor investigated in any manner, making this the initial reporting on EVs from this microorganism. This report details how we isolated EVs from *O. formigenes* culture supernatant by EV-specific affinity chromatography, confirmed and characterized these vesicles by nanoparticle tracking analysis (NTA), and analyzed the resulting EV isolate by PSI-MS using the Prosolia Velox 360 PSI source coupled to a Q Exactive Orbitrap MS. The novelty of this work is three-fold in that it demonstrates, to our knowledge, the first (1) PSI-MS analysis of EVs from any organism, (2) application of PSI-MS to bacterial exometabolomics, and (3) confirmation and investigation of EVs produced by *O. formigenes*. We believe this novel application of PSI-MS will serve the fields of metabolomics, exposomics, and analytical microbiology by providing a new platform for examining the chemical profile of EVs and the bacterial exometabolome.

## 2. Results and Discussion

### 2.1. Nanoparticle Tracking Analysis Confirms O. formigenes Extracellular Vesicles

**Figure 1:**
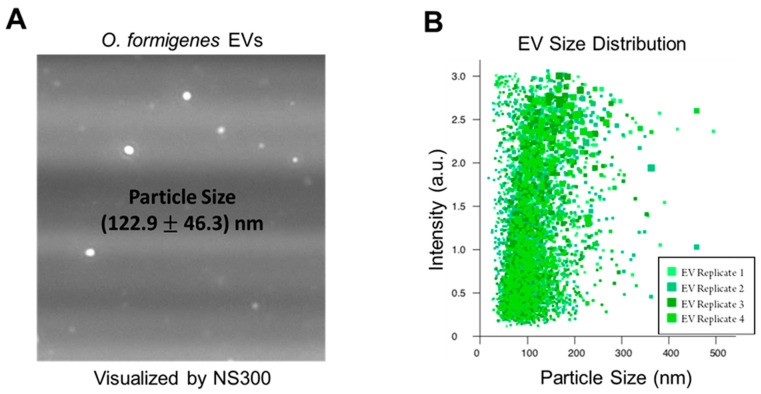
Nanoparticle Tracking Analysis confirms EVs in vesicle isolate from *O. formigenes* culture supernatant. (**A**) Image of EVs in purified isolate derived from *O. formigenes* culture supernatant captured by nanoparticle tracking analysis. (**B**) Particle size distribution of EVs detected in *O. formigenes* culture medium supernatant. Particle size (average ± standard error): (122.9 ± 46.3) nm, Particle size distribution: D_10_ = 80.4 nm, D_50_ = 111.5 nm, and D_90_ = 182.6 nm.

The production of EVs by *O. formigenes* in vitro was confirmed by NTA of our EV isolate from the bacterium’s culture supernatant. Figure 1A depicts an image of EVs from the *O. formigenes* isolate captured by the NanoSight NS300 (Malvern Panalytical, Malvern, United Kingdom). Vesicle size (average ± standard error) was measured as (122.9 ± 46.3) nm, which is consistent with the reported range for bacterial EVs [29,33], with D10 = 80.4 nm (meaning 10% of detected vesicles measured <80.4 nm), D50 = 111.5 nm, and D90 = 182.6 nm (Figure 1B). It is important to note that due to the fact that EVs are secreted for a variety of functions, their contents and characteristics, including particle size, could possibly be dependent on the specific environmental conditions the bacteria are experiencing and to which they are responding [55]. Therefore, the EV particle size (and size distribution) reported in this work should be taken only as a general reference as the effect of different media conditions, biotic and abiotic stressors, and other factors on the *O. formigenes* EV profile have not been investigated. Nevertheless, this serves as the first confirmation that *O. formigenes* produces these vesicles, and further work is needed to understand the specifics of their biological nature.

### 2.2. Extracellular Vesicle Metabolomics by PSI-MS

**Figure 2:**
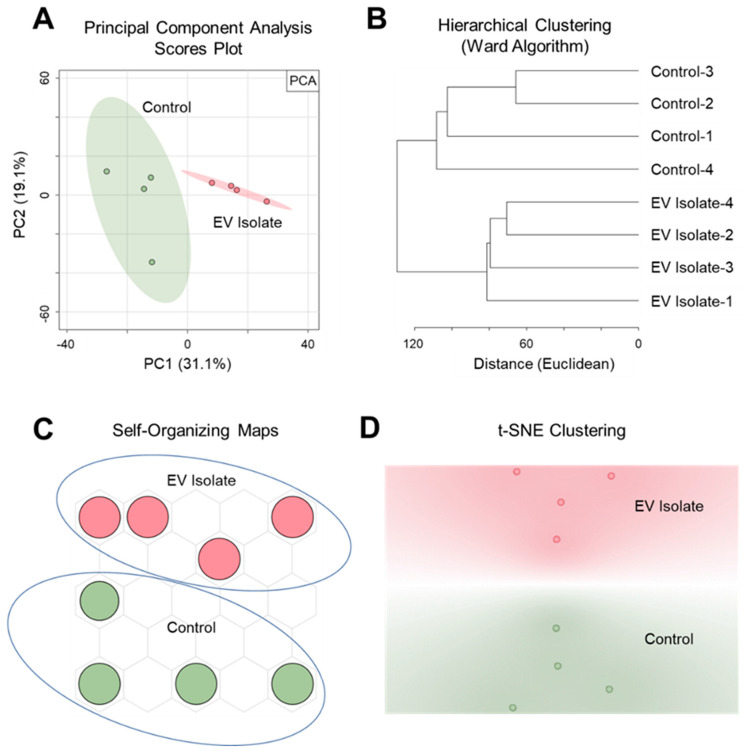
Unsupervised multivariate statistical clustering differentiates metabolomic profiles of the *O. formigenes* EV isolate and EV-free control. Clear separation and analytical distinction between the EV isolate and control was demonstrated using (**A**) principal component analysis (50.2% of the variance represented in 2 PCs), (**B**) hierarchical clustering (Euclidean distancing, Ward clustering), (**C**) self-organizing maps, and (**D**) t-stochastic neighbor embedding (initialized with PCA pre-processing).

PSI-MS analysis was successful both in distinguishing the EV isolate from an EV-free control as well as detecting a profile of vesicle-derived biochemical features. Here, we show our findings at the level of both general trends and specific metabolites. Our discussion focuses mainly on features that were exclusively detected in the EV isolate since this report is a demonstration of the ability of PSI-MS to detect vesicle metabolites rather than a comprehensive profiling of the EV metabolome. To exhibit the capability of PSI-MS to analytically differentiate the EV isolate from an EV-free control, we performed four different unsupervised statistical clustering analyses on the whole-metabolome dataset. Using four independent multivariate statistical approaches—principal component analysis (PCA) (Figure 2A), hierarchical clustering (Figure 2B), self-organizing maps (Figure 2C), and t-distributed stochastic neighbor embedding (Figure 2D)—the global metabolomes of the EV isolate and control were clearly separated due to significant metabolomic differences in their detected chemical profiles, indicating the presence of EV-derived features. From this point, these EV features became the primary focus of our analysis. The data were filtered for features that were exclusively detected in the EV isolate and absent in the EV-free control. In total, we detected and putatively identified 50 EV-derived features, details for which are provided in Table 1. Identifications, all of which we report as Level 2 in accordance with the metabolomics standards initiative (MSI) [56], were made by MS1 accurate *m/z* matching (≤5 ppm) to the METLIN database [57], focusing search results on expected ions/adducts (e.g. [M + H]^+^, [M + H − H_2_O]^+^, [M + Na]^+^) of known and biologically relevant compounds. The returned database matches show a high level of diversity in the detected *O. formigenes* EV chemical profile with metabolite, lipid, and peptide representation. Here we discuss the potential biological implications of a selection of these EV features. Most of the EV features were identified as lipids and small peptides. This was somewhat expected since a major contributor to the signal from this sample would likely be from the vesicle membrane, which would represent the bacterial membrane (rich in lipids and proteins) from which it originated [30].

Regarding lipids, we detected representative species from several major classes, including (among others) phosphatidylethanolamines (PE (37:5)), PE (38:1)), phosphatidylglycerols (PG (28:2), PG (36:5), PG (37:5)), phosphatidic acid (PA (41:7)), phosphatidylinositol (PI (35:0)), and phosphatidylserine (PS (41:0)). Detection of these specific lipids is supported by the fact that the membranes of Gram-negative bacteria are largely composed of various phospholipids, particularly PEs [58]. Furthermore, our previous work profiling the lipidome of *O. formigenes* HC1 corroborates these results by showing detection of most of these same lipid classes [59]. Nearly 50% of the EV features we detected were small peptides, mostly of 2–4 amino acid residues. As with lipids, significant detection of peptides is expected since many will originate from the bacterial membrane [30]. Small peptides, particularly dipeptides, have been shown to play important roles in cell signaling [60,61,62], meaning the peptides detected in this study could serve in various capacities in cellular communication and metabolism. One example is polyglutamic acid, which has been reported to be produced outside the cell by several species of bacteria, including both Gram-negatives and Gram-positives, and is believed to have multiple potential functions ranging from survival to virulence [63].

A variety of small molecule metabolites (non-lipid, non-peptide) were also detected in the EV isolate. While many of the metabolites we detected are expected components of conventional metabolism, our discussion will focus on two that are known to be primarily associated with bacteria and have suggested connections to human health: phenylacetylglutamine (PAG) and violacein. PAG is a gut microbiome-derived metabolite formed from the conjugation of glutamine and phenylacetate primarily by colonic microbial metabolism [64]. It has been proposed to serve as a biomarker for the progression of chronic kidney disease (CKD) due to the association observed between increased serum PAG levels and advanced-stage CKD [65]. Violacein is a pigment compound known to be produced by a variety of Gram-negative bacteria [66,67]. It is associated with a wide scope of biological functions, including having antibiotic [68], antiviral [69], anti-inflammatory [70], antifungal [66], and antitumor [67] properties, among many others [71]. Hence, there is significant interest in bacteria that produce this compound due to its potential impact on human health. While *O. formigenes*, to our knowledge, has not been shown to exhibit the purple hue typically seen in bacteria that produce violacein at appreciable levels [66], it is possible that it is expressed in low abundance sufficient to deliver its intended biological effects but without producing a visible purple tint in culture. The presence of PAG and violacein in *O. formigenes* EVs supports the notion that vesicles from this microorganism delivered in the gut could influence the health of the human host as part of the microbiome-derived exposome. Hence, further work is needed to confirm the identification and biological function of these secreted biochemicals to clarify the EV-mediated host-microbe relationship.

## 3. Materials and Methods

### 3.1. Isolation of O. formigenes Extracellular Vesicles from Culture Supernatant

EV isolation from *O. formigenes* supernatant was performed using the ExoBacteria OMV Isolation Kit (System Biosciences, Palo Alto, CA, USA) using the following process. It is important to note that although the name of the kit suggests it is specific to OMVs, we confirmed with the manufacturer that it does not discriminate between specific subtypes of bacterial EVs in its isolation (in the case of this analysis, between OMVs and O-IMVs). Hence, it captures all bacterial EVs in the final purified isolate. The compositions of all reagents and buffers in the kit (EV binding resin, EV binding buffer, EV elution buffer) were proprietary and undisclosed by the manufacturer. *O. formigenes* (strain HC1, a human isolate) was cultured from frozen glycerol stock in modified *Oxalobacter* medium (containing 100 mM oxalate; derived from DSMZ-German Collection of Microorganisms and Cell Cultures Reference Medium 419) in 100 mL anaerobic bottles by combining 4 mL glycerol stock with 76 mL media for a 5% *v*/*v* inoculum. A control medium (uninoculated) was carried in-parallel and identically handled through all subsequent steps of this procedure for downstream comparative PSI-MS analysis. We refer to this as the “EV-free control.” After incubating at 37 °C for 24 h, a 5% *v*/*v* subculture was generated in the same manner and allowed to incubate for 24 h. From this subculture, 80 mL turbid *O. formigenes* culture was harvested, transferred to clean 50-mL PP vials (40 mL in each of 2 vials), and centrifuged at 5000× *g* for 20 min at 4 °C to remove bacterial cells. After pelleting the bacteria, supernatants were transferred to new 50-mL PP vials and again centrifuged at 5000× *g* for 20 min at 4 °C. Supernatants were removed, filtered using a 0.22 μm syringe filter to ensure complete removal of bacterial cells, and transferred into new 50-mL PP vials. An EV affinity chromatography binding column was prepared by adding 1 mL EV binding resin stationary phase and washing with 10 mL EV binding buffer for equilibration. After sufficient washing and allowing the binding buffer to completely flow through the column, the bottom of the column was sealed, and 20 mL supernatant was added. The top of the column was then sealed, and the unit was placed on a rotating rack for 30 min at 4 °C to allow for mixing and EV binding to the resin. After 30 min, the top and bottom of the column were unsealed, and the supernatant was allowed to flow through the resin. This was repeated 2 additional times so that a total of 60 mL of culture supernatant, in 3 rounds of 20 mL, was allowed to mix with the resin on the rotating rack for 30 min at 4 °C for enhanced EV binding. After the third round of EV binding, the supernatant was allowed to flow through the column, and the resin was flushed with 45 mL EV binding buffer. Following the flush, the bottom of the column was sealed, and 750 μL EV elution buffer was added. Columns were allowed to incubate at room temperature for 2 min with gentle agitation every 30 s, after which the bottom of the column was unsealed, and 750 μL eluent containing the EV isolate was collected in a 1.5 mL PP vial. Samples were aliquoted and frozen at −80 °C until needed for analysis.

### 3.2. Nanoparticle Tracking Analysis of O. formigenes Extracellular Vesicles

NTA is a commonly used analytical technique for the detection and measurement of EVs, which, among other functions, observes the rate of Brownian motion of nanoparticles in an aqueous solution and relates this information to particle size [72]. The EV isolate was analyzed by NTA using the NanoSight NS300 (Malvern Panalytical) by the University of Florida Interdisciplinary Center for Biotechnology Research Cytometry Core. Analysis parameters reported by the core are provided in Table S1.

### 3.3. PSI-MS Instrumentation, Methodology, and Analysis

Velox cartridges containing pre-cut triangular paper (Prosolia Inc., Indianapolis, IN, USA) were deposited with 15 µL of EV isolate or EV-free control (n = 4 replicates per group). For this purpose, a 3D-printed pipette stabilizer (Prosolia Inc.) was used to ensure reproducibility in sample loading. Samples were analyzed using the Prosolia Velox 360 PSI source connected to a Q Exactive Orbitrap MS (Thermo Scientific, Waltham, MA, USA). The wetting and spray solvent was 7:3 water:acetonitrile with 0.1% formic acid (*v*/*v*/*v*). To the backside of the cartridge, 80 µL was dispensed in 8 sequential applications of 10 µL to elute the sample to the tip of the paper. Then, to the tip of the paper, 15 µL was dispensed in 5 sequential applications of 3 µL. Analysis was performed in full scan positive ion mode at 140,000 mass resolution for 30 s after 9 s equilibration. Scan range was *m/z* 70–1000, spray voltage was 4 kV, and capillary temperature was 270 °C. The S-lens was set to 30% to reduce source fragmentation.

### 3.4. Data Processing, Statistics, and Feature Annotation

File conversion from the native .raw format to the open-source .mzXML format was performed using RawConverter [73]. MZmine 2 was used for data processing, including mass detection, alignment, smoothing, deconvolution, isotope grouping, join aligning, gap filling, duplicate peak filtering, and removing adducts and complexes [74]. The resulting data were exported as a feature list containing the signal intensity of each detected feature (defined as a unique *m/z* value) in each sample. Features were designated as EV-specific by meeting 2 criteria: (1) if they were detected with a signal intensity ≥ 1 × 10^4^ in all EV isolate samples, and (2) if they showed no detection (signal intensity = 0) in any of the control samples. For multivariate statistical analyses only, which we performed using MetaboAnalyst 4.0 [75] and Orange Data Mining [76], half the minimum signal intensity value in the dataset was used to replace non-detected signals [77], and the data were normalized to total ion signal and autoscaled [78]. Putative metabolite identifications (MSI Level 2) were assigned using the METLIN database [57] based on accurate *m/z* matching (≤5 ppm) focusing search results on expected ions/adducts (e.g. [M + H]^+^, [M + H – H_2_O]^+^, [M + Na]^+^) of known and biologically relevant compounds.

## 4. Conclusions and Future Directions

In this report, we demonstrated a novel application of PSI-MS to the analysis of EVs by examining a bacterial EV isolate from *O. formigenes* culture supernatant, a bacterium whose EVs had never previously been investigated nor confirmed. We detected and putatively identified a panel of features deemed to originate from EVs by comparison to an EV-free control and observed representation from various classes of biochemicals, including metabolites, lipids, and peptides. From this work, we conclude that PSI-MS can serve as a new, rapid, sensitive, and economical approach to EV analysis. Our future endeavors to build upon the results from this investigation will mainly focus on the following: (1) confirming putative identifications assigned to EV features, which will require use of MS^2^/MS^n^ and comparison of fragmentation spectra to pure standards, and (2) broadening the scope of our analysis to a full characterization of the *O. formigenes* EV metabolome. Regarding the biological application of our results, we plan to evaluate EV-derived biochemicals for their potential impact on human health as part of the microbiome-derived exposome, particularly those that could participate in oxalate-regulating capacities (secretagogue candidates). Future work for the field in general should focus on optimization of PSI-MS parameters, particularly related to instrumentation, for analyzing EVs. A study examining the quantitative relationship between sample EV concentration and signal intensity and/or metabolome coverage to determine sufficient or optimal EV concentration for PSI-MS analysis would likely be of significant value. PSI-MS should be applied to a broad spectrum of EV experiments to examine the translatability of this analytical technique based on the unique types of EVs produced in different biological systems.

## Figures and Tables

**Table 1:** Features detected exclusively in *O. formigenes* EV isolate compared to an EV-free control. Putative IDs (MSI Level 2) made by accurate *m/z* matching to METLIN database (≤5 ppm).

*m/z*	Annotation	Molecular Formula (M)	Ion/Adduct	Δppm	METLIN ID
165.0153 ^a^	Muconic Acid	C_6_H_6_O_4_	[M + Na]^+^	3	45919
165.1016	Kynuramine	C_9_H_12_N_2_O	[M + H]^+^	3	43923
183.0261 ^a^	Oxoadipic Acid	C_6_H_8_O_5_	[M + Na]^+^	1	322
185.1280	Ala-Ile/Leu	C_9_H_18_N_2_O_3_	[M + H – H_2_O]^+^	5	8560(6/7)
196.0010	4-Phosphoaspartic Acid	C_4_H_8_NO_7_P	[M + H – H_2_O]^+^	0	360
205.1542	3-Hydroxy-N6,N6,N6-Trimethyl-L-Lysine	C_9_H_20_N_2_O_3_	[M + H]^+^	2	6324
213.0364 ^a^	4-Hydroxy-4-methyl-2-Oxoadipic Acid	C_7_H_10_O_6_	[M + Na]^+^	2	66102
213.1228	Pro-Pro	C_10_H_16_N_2_O_3_	[M + H]^+^	2	62027
218.1382	Propionylcarnitine	C_10_H_19_NO_4_	[M + H]^+^	2	965
227.1385	Hydroxyprolyl-(Iso)Leucine	C_11_H_20_N_2_O_4_	[M + H – H_2_O]^+^	4	8577(3/4)
229.1180	Prolylhydroxyproline	C_10_H_16_N_2_O_4_	[M + H]^+^	1	58518
241.1177	Gamma-Glutamyl-Pipecolic Acid	C_11_H_18_N_2_O_5_	[M + H – H_2_O]^+^	4	93275
246.0731 ^a^	Acetyltyrosine	C_11_H_13_NO_4_	[M + Na]^+^	2	5827
251.0522	Homocystine	C_8_H_16_N_2_O_4_S_2_	[M + H – H_2_O]^+^	0	4189
254.1378	3-Indolecarboxylic Acid	C_13_H_19_NO_4_	[M + H]^+^	3	6660
262.0851	Ser-Ala-Cys	C_9_H_17_N_3_O_5_S	[M + H – H_2_O]^+^	4	15654
265.1168	Phenylacetylglutamine	C_13_H_16_N_2_O_4_	[M + H]^+^	5	58397
295.2238 ^a^	Hydroxypalmitic Acid	C_16_H_32_O_3_	[M + Na]^+^	1	35428
297.0483	5′-Phosphoribosyl-N-Formylglycinamide	C_8_H_15_N_2_O_9_P	[M + H – H_2_O]^+^	2	3443
311.1456 ^a^	Arg-Asn	C_10_H_20_N_6_O_4_	[M + Na]^+^	5	23959
317.1929	Ala-Arg-Ala	C_12_H_24_N_6_O_4_	[M + H]^+^	0	21376
326.0909	Violacein	C_20_H_13_N_3_O_3_	[M + H – H_2_O]^+^	5	C21136 ^b^
337.1605	Ala-Gln-His	C_14_H_22_N_6_O_5_	[M + H – H_2_O]^+^	5	16023
345.1875	Ser-Arg-Thr	C_13_H_26_N_6_O_6_	[M + H – H_2_O]^+^	3	16028
359.1690	Asp-Arg-Ser	C_13_H_24_N_6_O_7_	[M + H – H_2_O]^+^	3	17672
361.1965	Arg-Trp	C_17_H_24_N_6_O_3_	[M + H]^+^	4	23686
367.1084	Met-Cys-Asn	C_12_H_22_N_4_O_5_S_2_	[M + H]^+^	5	15764
385.3061	N-Palmitoyl Glutamine	C_21_H_40_N_2_O_4_	[M + H]^+^	0	75509
407.2034	Ser-Arg-Tyr	C_18_H_28_N_6_O_6_	[M + H – H_2_O]^+^	2	15751
415.2289	Gly-Lys-Asn-Pro	C_17_H_30_N_6_O_6_	[M + H]^+^	2	146911
421.2315	His-His-Lys	C_18_H_28_N_8_O_4_	[M + H]^+^	2	18791
431.2394	Phe-His-Lys	C_21_H_30_N_6_O_4_	[M + H]^+^	1	18657
441.1496	Cys-Met-Ser-Thr	C_15_H_28_N_4_O_7_S_2_	[M + H]^+^	5	115796
445.1208	Cys-Cys-Gly-Tyr	C_17_H_24_N_4_O_6_S_2_	[M + H]^+^	0	111999
473.3075	Ile/Leu-Lys-Asn-Val	C_21_H_40_N_6_O_6_	[M + H]^+^	1	162916
475.2862	Ala-Glu-Lys-Lys	C_20_H_38_N_6_O_7_	[M + H]^+^	2	104848
479.1988	Ala-Asp-His-His	C_19_H_26_N_8_O_7_	[M + H]^+^	1	104406
501.1806	Polyglutamic Acid	C_20_H_30_N_4_O_12_	[M + H – H_2_O]^+^	5	58212
657.3238	Gln-Arg-Trp-Trp	C_33_H_42_N_10_O_6_	[M + H – H_2_O]^+^	3	213457
663.4264	Phosphatidylglycerol (28:2)	C_34_H_63_O_10_P	[M + H]^+^	4	79745
670.5166	Phosphatidylethanolamine (38:1)	C_38_H_74_NO_7_P	[M + H – H_2_O]^+^	1	60361
674.5555	GlcCer(d18:0/14:0)	C_38_H_75_NO_8_	[M + H]^+^	1	53987
734.5109	Phosphatidylethanolamine (37:5)	C_42_H_74_NO_8_P	[M + H – H_2_O]^+^	2	60354
761.5136	Phosphatidic Acid (41:7)	C_44_H_73_O_8_P	[M + H]^+^	2	81674
765.5086	Phosphatidylglycerol (37:5)	C_43_H_75_O_10_P	[M + H – H_2_O]^+^	2	79015
769.5023	Phosphatidylglycerol (36:5)	C_42_H_73_O_10_P	[M + H]^+^	1	61870
835.5720	Phosphatidylinositol (35:0)	C_44_H_85_O_13_P	[M + H – H_2_O]^+^	2	80078
862.6525	Phosphatidylserine (41:0)	C_47_H_92_NO_10_P	[M + H]^+^	0	78139
958.3124	Pentaglutamyl Folic Acid	C_39_H_47_N_11_O_18_	[M + H]^+^	5	58426
960.3109	Tetradecanoyl-CoA	C_35_H_62_N_7_O_17_P_3_S	[M + H – H_2_O]^+^	0	3707

^a^ Peaks corresponding to protonated ion [M + H]^+^ (Δppm ≤ 5) also detected for this species. ^b^ KEGG ID (not in METLIN database). Amino acid sequence orders of peptides should be regarded as interchangeable.

## Supplementary Materials

The following are available online at https://www.mdpi.com/article/10.3390/metabo11050308/s1, Table S1. Parameters for Nanoparticle Tracking Analysis of *O. formigenes* EV Isolate.
